# Understanding health problems in people with extremely low health-related quality of life in Korea

**DOI:** 10.1038/s41598-022-07528-2

**Published:** 2022-03-08

**Authors:** Thi Xuan Mai Tran, Sanghee Lee, Chang-Mo Oh, Yoon Jung Chang, Hyunsoon Cho

**Affiliations:** 1 Department of Cancer Control and Population Health, National Cancer Center Graduate School of Cancer Science and Policy, Goyang, Republic of Korea; 2 Department of Preventive Medicine, School of Medicine, Kyung Hee University, Seoul, Republic of Korea; 3 Division of Cancer Control and Policy, National Cancer Control Institute, National Cancer Center, Goyang, Republic of Korea; 4 Division of Cancer Registration and Surveillance, National Cancer Control Institute, National Cancer Center, Goyang, Republic of Korea

**Keywords:** Public health, Quality of life, Cancer

## Abstract

Little is known about patients reporting extremely poor health-related quality of life (HRQoL). This study targeted population with inferior HRQoL and examined their problems experienced with HRQoL dimensions, and impacts of different morbidities on these problems. Data were obtained from a population-based survey in Korea. HRQoL was measured by EQ-5D questionnaire and low-HRQoL population was defined as individuals whose EQ-5D utility score was among the lowest 5% of total survey population. Logistic regression models were used to evaluate the impact of fifteen morbidities on HRQoL dimensions. Of 2976 low-HRQoL participants, females and low socioeconomic individuals were predominant. They experienced significantly more problems in all dimensions, with pain/discomfort, and mobility as the most frequently reported problems. Problems in HRQoL dimensions diverged according to diseases. Individuals with arthritis experienced more difficulties with mobility (aOR 2.62, 95% CI 1.77–3.87) and pain/discomfort (aOR 2.86, 95% CI 1.78–4.60). Stroke patients experienced more problems in self-care (aOR 2.24, 95% CI 1.59–3.15) and usual activities (aOR 1.87, 95% CI 1.11–3.14). Having two or more diseases was associated with worse outcomes in usual activities and increased risk of depression. Thus, efforts to improve status of low-HRQoL should be customized to fulfil unmet needs corresponding to various diseases, and depression prevention is needed for those with multimorbidity status.

## Introduction

Health-related quality of life (HRQoL) is acknowledged as a core health indicator useful in guiding health policies and has been used to evaluate the effects of chronic diseases and various treatments. Therefore, many governments have raised more attention toward promoting HRQoL as one of the initiative's overarching public health goals to enhance population health and well-being. Meanwhile, many developed countries, including Korea, face challenges in their rapidly aging population and an increasing number of people with morbidities or chronic conditions who report low quality of life. For example, recent statistics from the Organisation for Economic Co-operation and Development (OECD)^1^ show that Koreans reported a generally lower subjective well-being indicator score than the OECD average, suggesting lower life satisfaction. Thus, despite the overall economic growth in Korea, a certain population still experienced low or extremely low HRQoL. Identifying these particular populations and understanding their sociodemographic characteristics or what health problems are impairing conditions might help improve the overall HRQoL of the community.

Previous studies have examined the impact of several diseases or conditions on HRQoL^2–5^. For example, health behavioral risk factors, such as smoking or physical inactivity, are associated with an impaired HRQoL^6^. However, many studies on the relationship between morbidities and HRQoL primarily focus on one specific disease or condition, such as arthritis^2^, bone mineral density^3^, chronic kidney disease^4^, and its negative impact on HRQoL. These studies thus often lack an overview of the divergence of HRQoL problems across various morbidities. In addition, few studies have been focused on individuals who experienced low HRQoL and explored which problems contribute towards HRQoL deterioration in this particular low-HRQoL population. Therefore, the present study aimed to provide a broad view of how different diseases deteriorate HRQoL dimensions among people with low QoL.

The EuroQoL (EQ-5D) questionnaire is one of the most widely used preference-based instruments assessing HRQoL, with five dimensions: mobility function, self-care, usual activity, pain or discomfort, and anxiety or depression. Recently, there has been increasing awareness and efforts at various levels to measure HRQoL in Korea. In Korea, EQ-5D was measured in an annual nationwide health survey, namely the Korean National Health and Nutrition Examination Survey (KNHANES) since 2005^7^. Using this population-based survey, we first targeted those who reported having extremely low QoL and aimed to understand their demographic as well as morbidity characteristics. Second, we examined how morbidities negatively impact different HRQoL dimensions. Additionally, as previous evidence yields gender and socioeconomic inequalities in HRQoL in the Korean population^8,9^, we investigated whether inequalities exist among this low-HRQoL population.

## Results

### Characteristics of the low-HRQoL population

A total of 50,583 participants were included in our study population. Of those, 2,976 participants were classified as a low-HRQoL population. Of 2,976 subjects reporting low QoL, 58.6% were elderly (age > 65), 68.2% were female, 41.3% had low income, 68.7% had an elementary education, and the mean age was 65.2 (0.4) years old (Table 1). Only 16.8% of the low-HRQoL population were disease-free, compared to 61.4% among the general population. In the low-HRQoL population, 47.1% had one or two diseases, and more than one-third (36.1%) had three or more. Further, in the low-HRQoL population, hypertension was the most prevalent disease (47.0%), followed by arthritis (42.5%), diabetes (21.3%), and hyperlipidemia (19.7%). Meanwhile, among the total population, the highest percentage of patients were diagnosed with hypertension (16.8%), followed by arthritis (9.8%), hyperlipidemia (8.8%), and diabetes (6.5%). When asked about overall health status in the low QoL population, 18.9% answered that they had normal overall health status, while 73.5% responded that their health status was bad or very bad.

**Table 1:** Demographic and disease characteristics of the low-HRQoL population versus the total survey population.

Characteristics	Total population (*n* = 50,583)		Low-HRQoL population (*n* = 2976)	
	*n*	%ª	*n*	%ª
Age, mean (SE)ª	45.3 (0.13)		65.2 (0.39)	
Elderly (age > 65)	11,179	13.3	2015	58.6
**Gender**				
Male	21,404	49.1	852	31.8
Female	29,179	50.9	2124	68.2
**Income**				
Low	12,230	25.4	1114	41.3
Middle low	12,545	25.2	757	25.7
Middle	12,581	25.0	591	18.8
High	12,536	24.4	429	14.2
**Education**				
Elementary/lower	13,298	18.6	2194	68.7
Middle school	5513	9.8	319	12.1
High school	17,075	39.0	324	13.6
College/higher	14,606	32.6	119	5.6
**Morbidity status (yes)**				
Hypertension	11,130	16.8	1476	47.0
Hyperlipidaemia	5423	8.8	573	19.7
Stroke	1128	1.6	334	10.5
Myocardial infraction	453	0.7	96	3.1
Angina pectoris	909	1.2	183	5.4
Arthritis	6929	9.8	1389	42.5
Asthma	1638	2.9	283	9.0
Tuberculosis	2472	4.4	209	7.2
Depression	2108	3.7	362	12.8
Diabetes	4226	6.5	655	21.3
Thyroid disease	1853	3.0	163	5.3
Cancer	1861	2.8	202	6.7
Liver diseases	988	1.9	78	2.8
Renal failure	219	0.4	52	1.9
Chronic kidney disease	1687	2.2	353	10.4
**Number of diseases**				
None	27,173	61.4	418	16.8
One or two	18,201	31.3	1433	47.1
Three or more	5209	7.3	1125	36.1
**Overall health status**				
Very good/Good	17,401	35.5	239	7.6
Normal	22,406	46.1	524	18.9
Bad/very bad	10,767	18.4	2210	73.5

*HRQoL*, Health-related Quality of Life; *SE* Standard error.

ªWeighted values.

### Dimensions of HRQoL experiencing problems in low-HRQoL population versus total survey population

**Figure 1:**
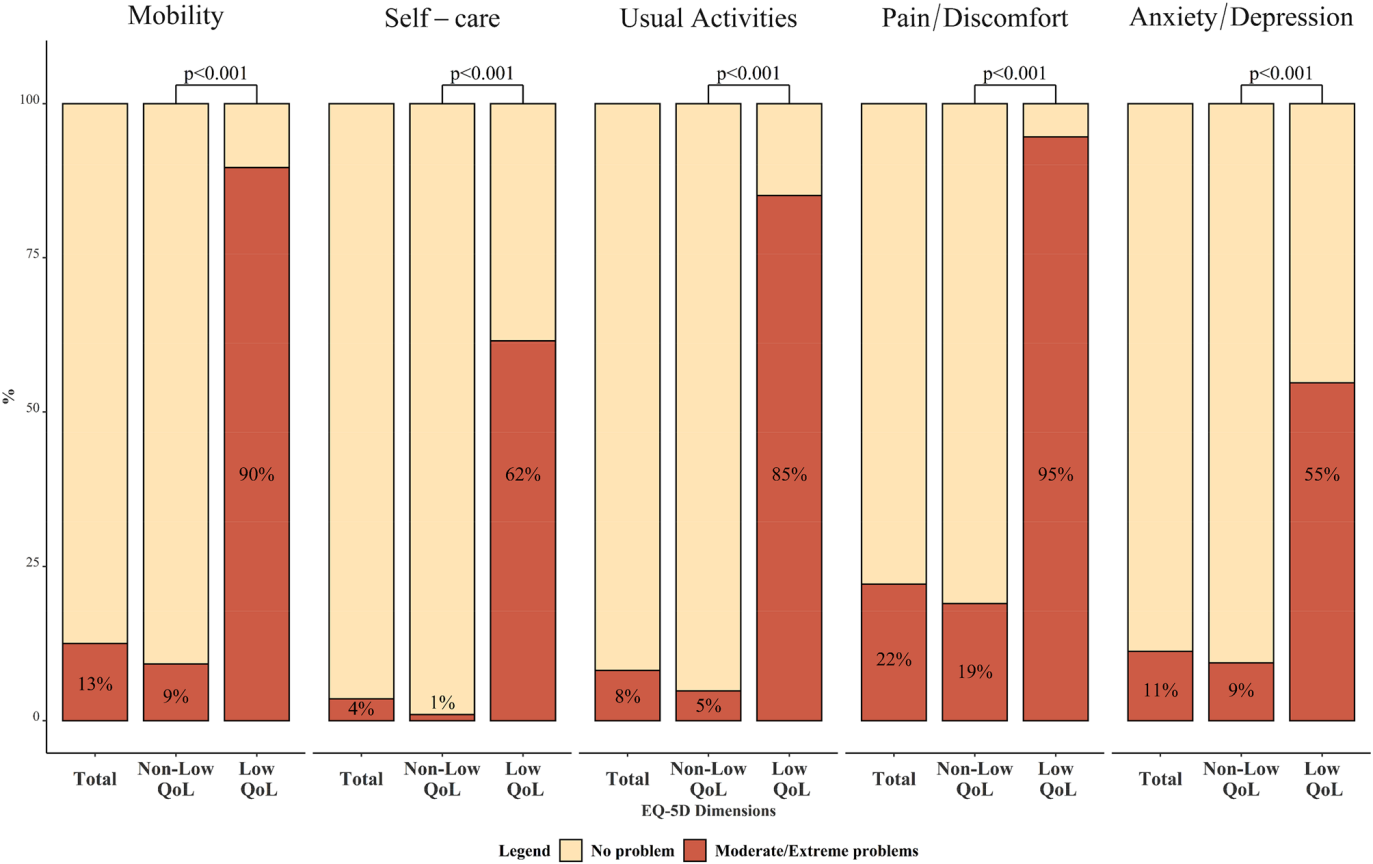
Proportions of individuals reporting moderate or extreme problems in HRQoL dimensions in the Total population, Non-Low-HRQoL, and the Low-HRQoL population. *P*-values were obtained from the Chi-square test to compare the proportion of reporting problems in EQ-5D dimensions.

The low-HRQoL population experienced more problems in all HRQoL dimensions than the total survey population (Fig. 1). Proportions of reporting problems were significantly higher in the low-HRQoL group than those of the rest of the survey respondents (non-low-HRQoL, all *p*-values < 0.001). More problems were noted in pain/discomfort (95% versus 19%) and mobility dimension (90% versus 9%). In terms of other dimensions, 85% of low-HRQoL populations reported having problems with their usual activities, 62% reported having self-care problems, and 55% experienced anxiety or depression.

### Divergence of HRQoL dimensions by morbidity

HRQoL dimensions diverged from disease to disease among the low-HRQoL population. Mobility problems were reported most frequently for some circulatory diseases, including stroke (95.4%) and arthritis (95.6%) (Table 2). Meanwhile, patients with hypertension, stroke, diabetes, and CKD reported more self-care problems than those without the disease. Stroke patients also reported significantly more problems in the usual activity dimension than the non-stroke population (93.3%, *p* < 0.001). More problems in the usual activity dimension were reported in patients with angina pectoris, asthma, and CKD. Arthritis patients experienced significantly more pain and discomfort than others (97.6%, *p* < 0.001, respectively).

**Table 2:** Moderate or extreme problems reported in the five HRQoL dimensions by disease status in the low-HRQoL population.

Disease	Mobility		Self-care		Usual activity		Pain/discomfort		Depression/anxiety	
	%ª	*p*^b^	%ª	*p*^b^	%ª	*p*^b^	%ª	*p*^b^	%ª	*p*^b^
**Hypertension**										
Yes	92.9	< 0.001	65.7	< 0.001	88.0	< 0.001	95.0	0.514	52.2	0.025
No	86.8		57.8		82.6		94.3		57.0	
**Hyperlipidemia**										
Yes	90.5	0.568	64.9	0.119	88.8	0.025	95.3	0.577	58.8	0.080
No	89.4		60.7		84.2		94.5		53.8	
**Stroke**										
Yes	95.4	0.006	79.4	< 0.001	93.3	< 0.001	92.7	0.174	52.9	0.554
No	89.0		59.4		84.2		94.9		55.0	
**Myocardial infarction**										
Yes	94.2	0.300	66.9	0.418	87.1	0.660	90.3	0.116	66.2	0.068
No	89.5		61.3		85.1		94.8		54.4	
**Angina pectoris**										
Yes	95.2	0.070	68.1	0.116	92.1	0.013	96.5	0.386	55.5	0.862
No	89.3		61.1		84.7		94.5		54.7	
**Arthritis**										
Yes	95.6	< 0.001	62.6	0.400	86.6	0.137	97.6	< 0.001	58.8	0.456
No	85.2		60.7		84.0		92.4		55.5	
**Asthma**										
Yes	89.4	0.931	62.9	0.683	90.3	0.040	95.1	0.786	57.7	0.383
No	89.6		61.4		84.6		94.6		54.5	
**Tuberculosis**										
Yes	89.5	0.973	59.6	0.610	90.0	0.082	93.3	0.536	55.2	0.915
No	89.6		61.7		84.8		94.7		54.7	
**Depression**										
Yes	82.8	< 0.001	53.9	0.012	85.7	0.790	94.4	0.897	82.8	< 0.001
No	90.6		62.6		85.0		94.7		50.6	
**Diabetes**										
Yes	92.8	0.022	69.0	< 0.001	86.3	0.447	92.8	0.063	52.6	0.310
No	88.8		59.5		84.8		95.1		55.3	
**Thyroid disease**										
Yes	95.4	0.022	49.5	0.007	84.2	0.778	98.0	0.028	61.8	0.131
No	89.3		62.2		85.2		94.4		54.4	
**Cancer**										
Yes	92.1	0.320	63.1	0.701	85.7	0.850	94.8	0.925	53.4	0.745
No	89.4		61.4		85.1		94.6		54.8	
**Liver diseases**										
Yes	84.9	0.268	54.0	0.255	72.1	0.011	95.4	0.784	56.1	0.847
No	89.8		61.7		85.5		94.6		54.7	
**Renal failure**										
Yes	87.6	0.721	60.1	0.870	83.5	0.823	91.9	0.511	53.8	0.911
No	89.7		61.5		85.2		94.7		54.8	
**Chronic kidney disease**										
Yes	94.4	0.021	69.3	0.008	90.1	0.025	94.2	0.755	51.9	0.329
No	89.1		60.6		84.5		94.7		55.1	

^a^Proportion of reporting problems (either moderate or severe) in each HRQoL dimension by disease status, weighted values.

^b^*p*-values were obtained from the Chi-square test to compare the proportion of reporting problem between those with and without having a disease.

**Figure 2:**
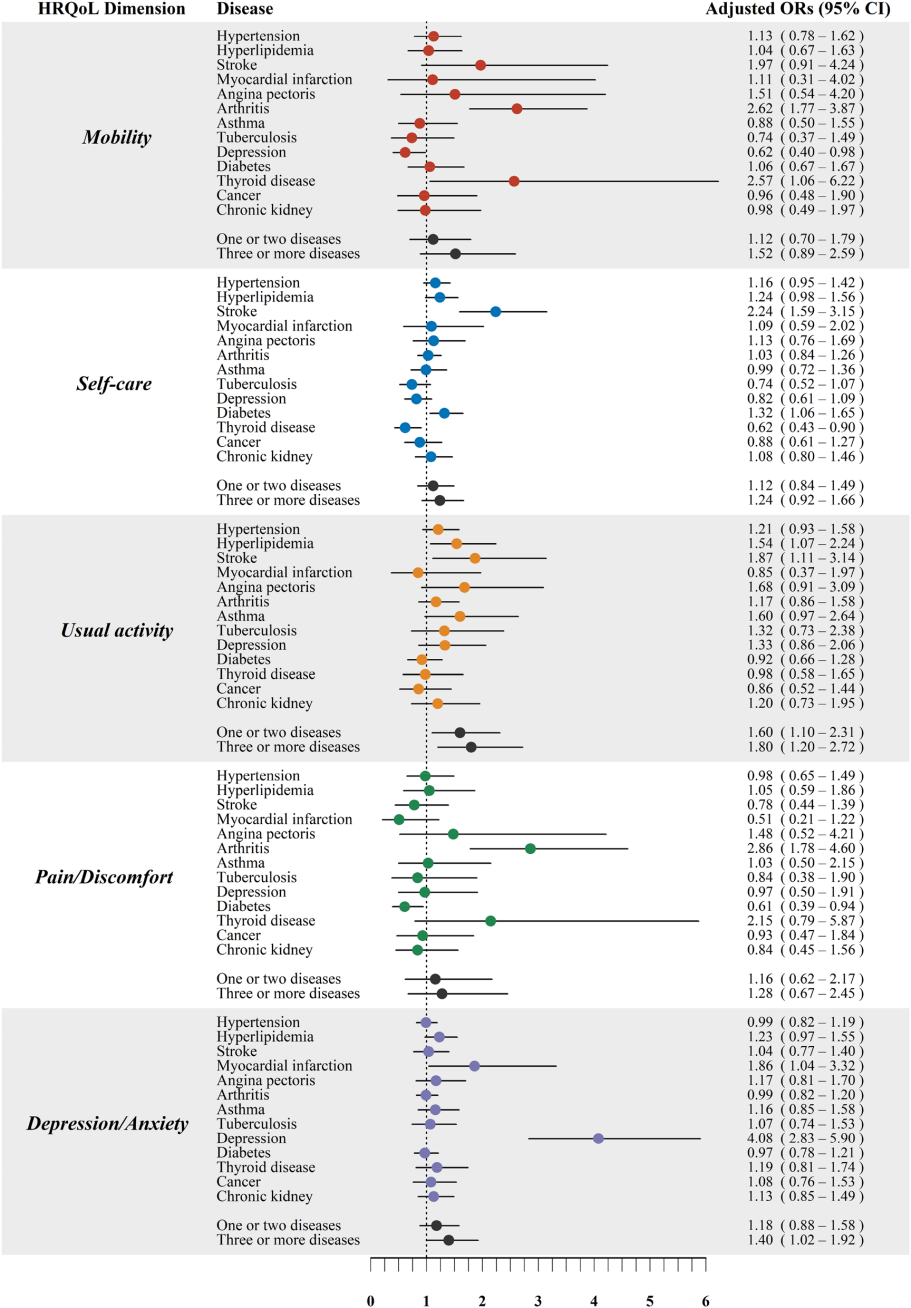
Multivariate logistic regression analysis of reporting problems in five HRQoL dimensions by different morbidities. Models were fitted separately for each HRQoL dimension and adjusted for age, gender, income level, and education level. The outcome variable in the logistic regression model was defined as "reporting no problem" versus "reporting problem" (either moderate or extreme problems).

After adjusting for confounding covariates, the multivariate logistic regression models estimate how dimensions are significantly associated with the diseases (Fig. 2). The odds of reporting problems in the HRQoL dimensions varied by disease type. Individuals with stroke reported higher odds of experiencing self-care (aOR 2.24; 95% CI 1.59 to 3.15) and usual activity (aOR 1.87; 95% CI 1.11 to 3.14) problems. Diabetes patients experienced significantly more self-care problems (aOR 1.32; 95% CI 1.06 to 1.65). Individuals with a thyroid disorder (aOR 2.57; 95% CI 1.06 to 6.22) and arthritis (aOR 2.62; 95% CI 1.77 to 3.87) experienced significantly more mobility problems. People with arthritis also showed higher odds of experiencing pain/discomfort (aOR 2.86; 95% CI 1.78 to 4.60).

The impact of multimorbidity on HRQoL among the low-HRQoL population was also evaluated (Fig. 2). Our results indicate that when participants had multimorbidity, they experienced significantly more usual activity problems: one or two disease conditions (aOR 1.60; 95% CI 1.10 to 2.31) and three or more comorbidities (aOR 1.80; 95% CI 1.20 to 2.72). Risk of having depression or anxiety also increased when participants had three or more comorbidities (aOR 1.40; 95% CI 1.02 to 1.92).

## Discussion

Recently, health promotion has expanded from focusing on mortality and morbidity to incorporating interventions that enhance the community's HRQoL. This article contributes to society health literature by exploring the population who experienced extremely low HRQoL and, further, better understanding their problems in disease-comparative settings. The explanatory power of our findings is relatively high since the analysis was based on nationwide data, and it corroborates previous results for this specific population. Some unique findings were gained from both their demographic and disease-characteristic perspectives. Problems with mobility function and pain/discomfort were most frequently reported in the low-HRQoL population relative to the general population. Problems in HRQoL dimensions are also diverged by the disease. A higher number of coexisting disease conditions were associated with limitations in their usual activities and increased the risk of depression. Our results further suggest that disparities in gender and socioeconomic status existed in the low-HRQoL population.

In this study, two of every three low-HRQoL people were female and had the lowest education level. Lower socioeconomic status and female gender thus exacerbate the risks of poor HRQoL outcomes; this "social gradient" in HRQoL is also evident in the previous studies^9,10^. Our results also indicate that the elderly population is dominant in the low-HRQoL population, accounting for approximately 80%. As age increases, the elderly might experience frailty, not only in their physical conditions but also in their psychological functions. Aging correlates with poorer HRQoL outcomes^8,9,11^. A notable distribution of elderly in the low-HRQoL population found in this study suggests that societal strategies to promote the status of these elderly are needed.

In the low-HRQoL population, pain and discomfort are the most frequently reported problems, consistent with previous evidence^12–15^. However, the impact on HRQoL dimensions differed by disease. Some specific patterns in how different diseases deteriorate HRQoL dimensions were explored. Stroke patients experience more problems with self-care and usual activities than the non-disease group. Meanwhile, individuals with arthritis suffered more in terms of mobility and pain/discomfort. Arthritis patients usually suffer from significant pain and disability, deteriorating their HRQoL^16–18^. Thyroid disorder patients experienced more difficulties in daily activity functions^19^. Thyroid disorders are rarely life-threatening; however, they can diminish patients' HRQoL because of the thyroid's essential roles^20^.

Depressive disorders are frequently comorbid with long-standing chronic conditions such as hypertension, diabetes, and cardiovascular diseases^21,22^. An increasing number of these coexisting diseases correlate with a higher risk of depression and consequently worsen HRQoL^23–25^, as found in this study. Depression and anxiety might cause barriers to a treatment course for the comorbidities and therefore worsen health outcomes^26^. Even though patients with multi-comorbidity may experience lower HRQoL than others, they may not receive the attention they deserve^27,28^. Our findings highlight that individuals with multiple disease conditions, particularly depression and anxiety, should be prioritized to diminish disease burden and improve overall HRH.

The EQ-5D used in this study has national social tariffs, also called a value set, derived from the Korean population^29^. However, concerns have been raised about the ceiling effects of the EQ-5D when used in general populations^30,31^. A high proportion of the perfect scores in HRQoL dimensions have resulted in high mean EQ-5D indexes in different populations^14^. This skewed nature of the EQ-5D scores causes a lack of discrimination among mild health states when analyzing HRQoL data from KNHANES. Given this phenomenon, we aimed to target only those who reported the lowest 5% of EQ-5D utility scores in this study and considered them as the low-HRQoL population. We believed that this approach might result in better discrimination of low- and non-low-HRQoL populations.

As abovementioned, the current study aimed at the participants who reported extremely low quality of life, and thus, the average EQ-5D index score was 0.72, which is substantially lower than the average score of 0.96 of the total KNHANES study population^32^. The EQ-5D index value of 0.72 thus indicates a disparity in people with extremely low HRQoL relative to the general population. In addition, the recent statistics indicate that the EQ-5D index decreased considerably with increasing age, and a gender gap, with worse HRQoL in females, was reported. Therefore, future health care policy or welfare programs are needed to shorten the gap in HRQoL between the low HRQoL and the general population, with additional consideration of the inequalities arising from age and gender.

Two main points should be explicitly acknowledged and considered when interpreting the results of our study. First, since KNHANES is meant for non-hospitalized civilians only, persons with severe conditions were likely to be excluded. Therefore, for some diseases well known to lead to low HRQoL, such as stroke or cancer, as measured in this survey, HRQoL status might be underestimated. Other limitations of our study should also be acknowledged. For example, KNHANES only includes the 15 most common disease types, whereas different disease types or external factors might impact this low-HRQoL population.

In general, the low-HRQoL population endures the most pain, discomfort, and mobility function issues, emphasizing the need for a more comprehensive approach concentrating on these problems. In addition, those with more coexisting diseases might benefit from interventions to prevent depression. Since the impact on HRQoL dimensions diverged by disease, interventions to improve overall HRQoL should be customized to focus on related issues and fulfil their unmet needs. Additionally, women and those with lower socioeconomic status more frequently experienced lower HRQoL than others. Hence, HRQoL inequalities arising from gender and socioeconomic status merit further investigation and appropriate interventions.

## Method

### Participants

Data were obtained from the multi-year KNHANES from 2007 to 2015. The KNHANES is a nationwide population-based complex multistage survey. The participants were chosen by a complex proportional allocation system and systematic sampling with multistage stratification, age, sex, and region^7^. This study was approved by the National Cancer Center Institutional Review Board of Korea (Approval Number: NCC2018-0284), and was conducted in accordance to the guidelines of the Declaration of Helsinki-ethnical principles for medical research involving human subjects. All of the participants provided informed consent prior to participating in the KNHANES.

### Measures

#### Health-related quality of life measure

HRQoL was measured using EuroQoL Five-Dimension (EQ-5D). The five dimensions were measured by five related questions evaluating a participant's health status: mobility, self-care, usual activity, pain/discomfort, and depression/anxiety. Response levels were as follows: "no problem," "moderate problem," and "extreme problem." In this study, we re-categorized the three classes into two groups: "report no problem" and "report problem" (either moderate or extreme problems). The Korean version of the EQ-5D was cross-culturally adapted and validated in a previous study^33,34^. The kappa value of EQ-5D dimensions between test and retest was 0.32–0.64, and the intraclass correlation coefficient of the EQ-5D index was 0.61. Therefore, EQ-5D is considered a useful instrument for measuring HRQoL of the general Korean population, with a moderate convergent and discriminant validity^33,34^.

#### Low-HRQoL population

The EQ-5D index score was calculated using the Korean value set for this instrument^29^. The range of EQ-5D index scores was from −0.17 to 1. One indicates perfect health condition, zero indicates a condition as bad as death, and less than zero indicates a subjective condition worse than death^35^. We defined our target population as the lowest 5% of the EQ-5D index of the total KNHANES population (EQ-5D index score ≤ 0.72).

#### Morbidity status

Fifteen diseases were included in our analysis: hypertension, hyperlipidemia, stroke, myocardial infarction, angina pectoris, arthritis, pulmonary tuberculosis, asthma, cancer, diabetes mellitus, thyroid, depression, liver diseases (hepatitis B, hepatitis C, hepatocirrhosis), renal failure, and chronic kidney disease (CKD). Subjects were asked about their disease status for a range of diseases in the form of an adjustable question: for example, "Have you ever been diagnosed with stomach cancer by a doctor?" Those who answered "yes" were considered to have that disease. CKD disease was defined as a glomerular filtration rate less than 60 mL/min/1.73m^2^, calculated from creatinine level, in the health examination^36^. Details on each disease measurement were described in Supplementary Table 1.

#### Demographic factors

Sociodemographic factors included age, gender, income level, and education level. Income was categorized into four quartile household income groups: low (the lowest 25%), middle-low (between 25 and 50%), middle (between 50 and 75%), and high (the highest 25%). Education levels were categorized into elementary graduate or lower, middle school graduate, high school graduate, and college graduate or higher.

### Statistical analysis

Descriptive statistics were calculated to describe the participants' sociodemographic characteristics, and chi-squared tests were used to compare distributions in HRQoL problems. We fitted logistic regression models to evaluate the probability of individual reporting problems in each EQ-5D dimension by disease status. In the model, the non-disease counterparts were used as the reference group (e.g., cancer versus non-cancer). We fitted a model for each disease and adjusted for age, gender, income level, and education level. In our analysis, we considered the stratified multistage clustered probability sampling design and survey weights. All statistical analyses were performed using the SAS survey procedures in SAS software version 9.4 (SAS Inc., Cary, NC). *P*-values less than 0.05 were considered statistically significant.

## Supplementary Information

Supplementary Information.
