# Supplementary material for: Understanding health problems in people with extremely low health-related quality of life in Korea
Source: Sci Rep. 2022 Mar 8;12:4037. doi: 10.1038/s41598-022-07528-2 (PMC8904781; doi:10.1038/s41598-022-07528-2)
Supplement: Supplementary file 1 — Supplementary Information. [file 41598_2022_7528_MOESM1_ESM.docx]

**Supplementary Table 1. List of diseases included in the study and their definitions**

| **No.** | **Disease** | **Measurement method** |
| --- | --- | --- |
| 1 | Hypertension | Using self-reported questionnaire, those who responded “Yes” to the following question “Have you ever been diagnosed with **hypertension** by a doctor?” was considered as having the disease. |
| 2 | Hyperlipidemia | Using self-reported questionnaire, those who responded “Yes” to the following question “Have you ever been diagnosed with **hyperlipidemia** by a doctor?” was considered as having the disease. |
| 3 | Stroke | Using self-reported questionnaire, those who responded “Yes” to the following question “Have you ever been diagnosed with **stroke** by a doctor?” was considered as having the disease. |
| 4 | Myocardial Infarction | Using self-reported questionnaire, those who responded “Yes” to the following question “Have you ever been diagnosed with **myocardial infarction** by a doctor?” was considered as having the disease. |
| 5 | Angina Pectoris | Using self-reported questionnaire, those who responded “Yes” to the following question “Have you ever been diagnosed with **angina pectoris** by a doctor?” was considered as having the disease. |
| 6 | Arthritis | Using self-reported questionnaire, those who responded “Yes” to the following question “Have you ever been diagnosed with **arthritis** by a doctor?” was considered as having the disease. |
| 7 | Pulmonary Tuberculosis | Using self-reported questionnaire, those who responded “Yes” to the following question “Have you ever been diagnosed with **pulmonary tuberculosis** by a doctor?” was considered as having the disease. |
| 8 | Asthma | Using self-reported questionnaire, those who responded “Yes” to the following question “Have you ever been diagnosed with **asthma** by a doctor?” was considered as having the disease. |
| 9 | Cancer | Using self-reported questionnaire, those who responded “Yes” to the following question “Have you ever been diagnosed with **cancer** by a doctor?” was considered as having the disease.  Specific cancer type was surveyed, including gastric, liver, colorectal, breast, cervical and other cancers. Those who answered any of the abovementioned cancer types was considered as having cancer. |
| 10 | Diabetes Mellitus | Using self-reported questionnaire, those who responded “Yes” to the following question “Have you ever been diagnosed with **diabetes mellitus** by a doctor?” was considered as having the disease. |
| 11 | Thyroid | Using self-reported questionnaire, those who responded “Yes” to the following question “Have you ever been diagnosed with **thyroid** by a doctor?” was considered as having the disease. |
| 12 | Depression | Using self-reported questionnaire, those who responded “Yes” to the following question “Have you ever been diagnosed with **depression** by a doctor?” was considered as having the disease. |
| 13 | Liver Diseases (Hepatitis B, Hepatitis C, Hepatocirrhosis) | Using self-reported questionnaire, those who responded “Yes” to the following question “Have you ever been diagnosed with **hepatitis B/hepatitis C/hepatocirrhosis** by a doctor?” was considered as having the disease. |
| 14 | Renal Failure | Using self-reported questionnaire, those who responded “Yes” to the following question “Have you ever been diagnosed with **renal failure** by a doctor?” was considered as having the disease. |
| 15 | Chronic Kidney Disease | Using creatinine level measured in the health examination, and defined as a glomerular filtration rate less than 60 mL/min/1.73m^2^ |
